# Highly Loaded Independent Pt^0^ Atoms on Graphdiyne for pH‐General Methanol Oxidation Reaction

**DOI:** 10.1002/advs.202104991

**Published:** 2022-04-07

**Authors:** Lan Hui, Yurui Xue, Chengyu Xing, Yuxin Liu, Yuncheng Du, Yan Fang, Huidi Yu, Bolong Huang, Yuliang Li

**Affiliations:** ^1^ Institute of Chemistry Chinese Academy of Sciences Beijing 100190 P. R. China; ^2^ School of Chemical Sciences University of Chinese Academy of Sciences Beijing 100049 P. R. China; ^3^ Science Center for Material Creation and Energy Conversion School of Chemistry and Chemical Engineering Shandong University Jinan 250100 P. R. China; ^4^ Department of Applied Biology and Chemical Technology The Hong Kong Polytechnic University Hung Hom Kowloon Hong Kong SAR P. R. China

**Keywords:** atomic catalysis, fuel cells, methanol oxidation reactions, two dimensional graphdiyne

## Abstract

The emergence of platinum‐based catalysts promotes efficient methanol oxidation reactions (MOR). However, the defects of such noble metal catalysts are high cost, easy poisoning, and limited commercial applications. The efficient utilization of a low‐cost, anti‐poisoning catalyst has been expected. Here, it is skillfully used N‐doped graphdiyne (NGDY) to prepare a zero‐valent platinum atomic catalyst (Pt/NGDY), which shows excellent activity, high pH adaptability, and high CO tolerance for MOR. The Pt/NGDY electrocatalysts for MOR with specific activity 154.2 mA cm^−2^ (1449.3 mA mg_Pt_^−1^), 29 mA cm^−2^ (296 mA mg_Pt_^−1^) and 22 mA cm^−2^ (110 mA mg_Pt_^−1^) in alkaline, acid, and neutral solutions. The specific activity of Pt/NGDY is 9 times larger than Pt/C in alkaline solution. Density functional theory (DFT) calculations confirm that the incorporation of electronegativity nitrogen atoms can increase the high coverage of Pt to achieve a unique atomic state, in which the shared contributions of different Pt sites reach the balance between the electroactivity and the stability to guarantee the higher performance of MOR and durability with superior anti‐poisoning effect.

## Graphical abstract

Independent zero‐valent Pt atoms on N‐doped GDY to prepare a zero‐valent platinum atomic catalyst (Pt/NGDY) are skilfully anchored. The results reveal that the inhomogeneous electronic distribution of NGDY allows the dispersive and high coverage of Pt atoms, boosting up the electroactivity for efficient methonal oxidation reaction (MOR). The Pt/NGDY electrocatalysts exhibit higher specific activity for MOR than commercial Pt/C.

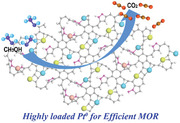

## 1 Introduction

Because of its unique and superior characteristics, atomic catalyst (ACs) has become one of the hottest research frontier fields in renewable energy conversion. This is mainly due to its ≈100% metal atom utilization, infinitely distributed and uniform active sites to achieve high catalytic selectivity, activity and stability in various sustainable energy technologies (e.g., fuel cells, batteries, and hydrogen production devices, etc.).^[^ ^1^, ^2^, ^3^, ^4^, ^5^, ^6^, ^7^, ^8^, ^9^ ^]^ Previous reports have shown that the unique atomic environments of the active sites (for example, geometric construction, coordination, and electronic structure) in ACs are decisive in determining the catalytic efficiency.^[^ ^10^, ^11^, ^12^ ^]^ More importantly, the special geometric and electronic structures of ACs allow for the modulation of the binding behaviors of reaction intermediates, which can lead to different reaction selectivity, activity, and stability in catalytic processes.^[^ ^3^, ^4^, ^13^ ^]^ Accordingly, the configurations of ACs can be further exploited to tune the catalytic performances toward target reactions. A very important issue is to rationally design and synthesize ACs with desired atomic environments aiming to enhance the conversion efficiencies toward practical applications.

Direct methanol fuel cells (DMFC) have been considered one of the most promising energy conversions with high energy density,^[^ ^14^, ^15^, ^16^ ^]^ in which Platinum‐based catalysts are employed as the most promising electrocatalysts for efficient methanol oxidation reaction.^[^ ^17^, ^18^ ^]^ Previously, great efforts have been devoted to enhancing the MOR performance through (1) fabricating the Pt‐M (M = Ni, Pd, Co, Ru) alloying with special structure^[^ ^17^, ^18^, ^19^, ^20^ ^]^; (2) designing Pt‐C (C = rGO, CNTs, NGO) catalysts dispersed on carbon support^[^ ^19^, ^21^, ^22^ ^]^; (3) Anchoring single‐atom on Pt nanostructure or metal oxidation.^[^ ^23^ ^]^ These strategies exhibit enhanced electrocatalytic activity and durability for MOR by tunning electronic structure, improving the dispersing on supports, or fabricating the atomic vacancies.^[^ ^14^, ^19^, ^23^ ^]^ However, the low kinetic activity and self‐poisoning are due to the intermediate CO adsorption on Pt leads to the rapid decrease in the catalytic performance. This is a difficult issue in the catalytic field. Well solved, it can well guide us to develop new and general electrocatalysts with high reaction efficiency and realize the rapid development of the energy industry. Hence, the challenge we face is how to develop efficient and general electrocatalysts with high resistance to CO poisoning, and high pH adaptability for MOR.

Graphdiyne, a new carbon allotrope comprising of sp/sp^2^‐hybridized carbon atoms, affords unique opportunities for rational elemental doping,^[^ ^24^ ^]^ for instance, sp—for nitrogen^[^ ^25^ ^]^—and sp^2–^hybridized carbon atoms for hydrogen^[^ ^26^ ^]^–, fluoro^[^ ^27^ ^]^–, chlorine^[^ ^28^ ^]^–, and boron.^[^ ^29^ ^]^ These new materials, developed on the basis of graphdiyne, endow graphdiyne with special properties for various applications including catalysis,^[^ ^24^, ^30^, ^31^, ^32^ ^]^ energy storage and conversion.^[^ ^33^, ^34^, ^35^ ^]^ N‐doped carbon supports have made a great contribution to the development of ACs due to their defect‐engineering to stronger chemical bonds, offer and stabilize more atomically sites.^[^ ^36^, ^37^, ^38^ ^]^ The N‐doped carbon SACs catalysts applied for different electrocatalytic including formic acid oxidation reaction, hydrogen evolution reaction and oxygen reduction reaction with high activity and stability in the nearest reports.^[^ ^39^, ^40^, ^41^ ^]^ The high electronegativity of N atoms induces more charge transfer between GDY support and low coordination metal ACs in this process, which results in the ACs on N‐doped GDY supports more stable and active.^[^ ^25^ ^]^ In addition, the N‐doping induced intrinsic defects, large surface area and abundant porous size of GDY supports favor anchoring more metal sites.^[^ ^42^ ^]^ Importantly, N‐doped GDY supports can exhibit high electric conductivity, excellent stability in acid and alkaline electrolytes.^[^ ^43^ ^]^ Encouraged by these excellent advantages of N‐doped GDY, we speculated the single atom Pt on NGDY with notable properties can enhance catalyze the MOR. Although the atomic catalysts have been widely applied in many other electrochemical reactions, Pt single‐atom catalyst on N‐doped GDY applied in the MOR have rarely been discussed.

Herein, we report the facile anchoring of zero‐valent Pt atoms on N‐doped graphdiyne obtained by selective cycloaddition of sp‐hybridized carbon atoms in GDY with hydrazine (Pt/NGDY). Experimental results showed that the Pt/NGDY has excellent activity, CO anti‐poisoning ability and durability for efficient MOR over a wide pH range from acidic to alkaline conditions. Our results revealed that the inhomogeneous electronic distribution induced by N dopants allows the dispersive and high coverage of Pt atoms, which boosts up the electroactivity based on more positively charged Pt to enhance the fixation of intermediates and electron transfer for MOR. This work paves a new direction for the atomic catalyst to achieve comparable performance with nanoparticles in the MOR through the high‐loading strategy.

## 2 Results and Discussion

**Figure 1:**
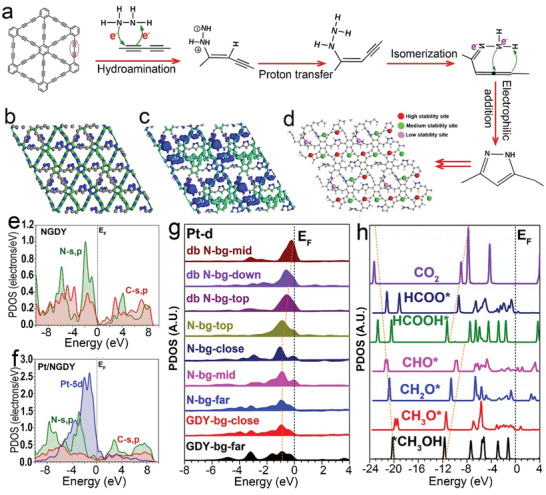
a) Schematic representation of the synthetic route for NGDY. b) Structural configurations and real spatial 3D orbital contour plots of NGDY. c) Structural configurations and real spatial 3D orbital contour plots of high coverage Pt/NGDY. d) The mapping of high coverage Pt/GDY. e) PDOSs of the high coverage NGDY. f) PDOSs of the high coverage Pt/NGDY. g) PDOSs of the Pt atom on different anchoring sites on NGDY. h) PDOSs of key adsorbates of MOR in Pt/NGDY.

Benefiting from the rich in diacetylene units, GDY allows for the precise and controllable cycloaddition reaction, which can result in a new type of pyrazole‐nitrogen doped GDY with accurate N‐doping sites. The Cope‐type hydroamination of diacetylenes in GDY with hydrazine was performed to form NGDY, as shown in **Figure** **1**a. In brief, the NGDY was synthesized by the selective cycloaddition of diacetylene in GDY with hydrazine, including the Cope‐type hydroamination of diacetylenes with hydrazine occurred together with a proton‐transfer, followed by a fast isomerization and an intramolecular electrophilic addition.^[^ ^5^ ^]^ Electrochemical in situ anchoring was performed through a chronopotentiometry method at the current density of 5 mA cm^–2^ for 10 s by a three‐electrode system, in which the self‐supported three dimension (3D) NGDY nanosheets array was used as the working electrode. The Pt atoms were seized and anchored on the NGDY nanosheets, achieving the single Pt atom catalysts, whereas longer deposition time (e.g., above 20 s) would dramatically result in Pt nanoparticles on NGDY nanosheets (Figure S1, Supporting Information).

Density functional theory (DFT) calculations were performed to explore the origins of high coverage of Pt atoms on the NGDY and their high performance in MOR. For the structure of NGDY, we have constructed the model with N dopants of different distributions in GDY. The bonding and antibonding orbitals near the Fermi level (*E*_F_) are demonstrated for the NGDY, which supports that the electron‐rich feature of the C sites and the N dopants on the chains of GDY (Figure 1b). This indicates both the structural symmetry and the electronic distribution has been affected by the N dopants, which creates more potential sites for the anchoring of Pt atoms to achieve high coverage and loading. With the sufficient coverage (≈70%) of Pt atoms on the NGDY, we notice the evident distortion of the local structure induced by the substantial *p‐d* couplings between the NGDY and Pt atoms (Figure 1c). More importantly, the local electronic structure has been further perturbed, leading to highly uneven electronic distribution, where different Pt sites have displayed different electronic contributions. For high coverage, we notice that the Pt atoms preferred to distribute in the lattice to reach high stability (Figure 1d). Regarding the energy cost for the anchoring, we have classified the various Pt sites into three different types based on the energy. Notably, the Pt atom anchored on the GDY chain without N dopants still demonstrates the highest stability with the lowest energy cost. As the neighboring N dopants become more, the anchored Pt atoms are less stable with a high energy barrier to be stabilized. To reveal the electronic structure induced by the N dopants and Pt, we have compared the projected partial density of states (PDOS) of NGDY and Pt/NGDY (Figure 1e). For the NGDY, we notice the higher position of N‐*s,p* orbitals than the C‐*s,p* orbitals. A minor gap is still noticed between the conduction band (CB) and valence band (VB), indicating the barrier for electron transfer. With the high coverage of Pt atoms, the electronic structure has been evidently changed (Figure 1f). The Pt‐5d orbitals dominate the electronic states near *E*_F_. Notably, the N‐*s,p* orbitals have been significantly suppressed from *E*_V_−1.94 eV in NGDY to the slightly deeper position at *E*_V_−2.90 eV (*E*_V_ = 0 eV). The *s,p* orbitals of GDY show the broadband feature and cross the Fermi level, indicating the improved electronic conductivity. This is attributed to the *p‐d* coupling by the high coverage of Pt. Since we have noticed different anchoring sites of Pt, the corresponding site‐dependent electronic structures of anchored Pt atoms have been investigated (Figure 1g). For those highly stable anchoring sites on the GDY chain without N dopants involvement, the dominant peak of 5d orbital in Pt is located near *E*_V_−1.94 eV. The consistent results with XPS data of experiments confirm that the high coverage of Pt atoms in NGDY consists of varied types of anchoring sites, which achieves the balance between stability and electroactivity. To evaluate the MOR performance, the PDOS of the key intermediates has been demonstrated (Figure 1h). Owing to the contribution of different Pt sites, the PDOS of the intermediates displays the linear correlation, which supports the efficient electron transfer during the MOR process. The much‐increased active Pt sites on NGDY enable the intermediate transformations simultaneously on varied neighboring Pt sites, which significantly boosts up the MOR process and guarantees the high performance.

**Figure 2:**
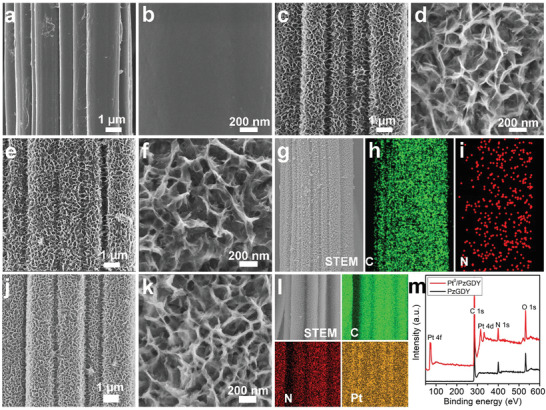
Morphological characterizations. Low‐ and high‐magnification SEM images of a,b) pure CC, c,d) GDY, and e,f) NGDY, respectively. g) SEM images and elemental mapping images of h) C and i) N. j) Low‐ and k) high‐magnification images of Pt/NGDY, l) SEM and corresponding elemental mapping images of C, N, and Pt, respectively. m) XPS survey spectra of NGDY (black line) and Pt/NGDY (red line).

Scanning electron microscopy (SEM) images in **Figure** **2**a–d show that the GDY nanosheets array was uniformly grown on carbon cloth with a smooth surface (Figure 2a,b), forming a honeycomb‐type porous structure. During the cycloaddition process, the morphology of GDY was well maintained (Figure 2e,f) and the N elements were distributed uniformly over the whole nanosheets (Figure 2g–i). From SEM (Figure 2j,k) and transition electron microscopy (TEM, Figure S2, Supporting Information) images, we can see that the Pt/NGDY still retains the honeycomb‐type porous structure which is beneficial for the catalytic reaction process. Energy‐dispersive X‐ray spectroscopy (EDS) mapping (Figure 2l) showed the homogeneous distribution of Pt, N and C elements. X‐ray photoelectron spectroscopy (XPS, Figure 2m) revealed the successful anchoring of Pt and on NGDY.

**Figure 3:**
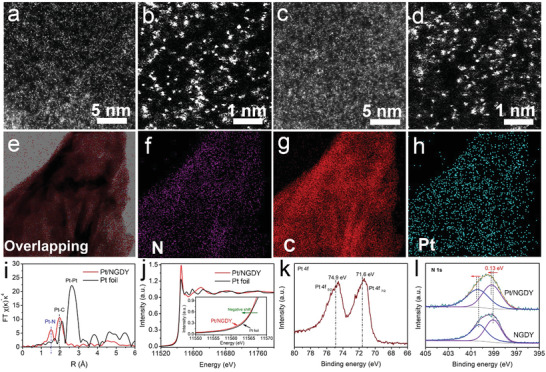
HAADF and structure characterizations of the sample. a,c) The HAADF images and b,d) enlarged images of the Pt/NGDY recorded from different areas (Bright dots are single Pt atoms). The e) STEM image and f) EDS elemental mapping images of overlapping, g) C and h) Pt, respectively. i) Fourier transform (FT) of the Pt L3 edge EXAFS spectra of Pt/NGDY and Pt foil. j) Pt L3‐edge spectra for Pt/NGDY and Pt foil. k) Pt 4f, l) N 1s and XPS spectra of the samples.

High‐angle annular dark‐field scanning transmission electron microscopy (HAADF‐STEM) was employed to characterize the atomic structure of the catalysts. The HAADF‐STEM images display large numbers of bright dots isolatedly and highly dispersed on the substrate surface and no peak of Pt was observed in XRD (**Figure** **3**a–h, Figure S3, Supporting Information), which confirmed the atomic dispersion of Pt atoms on NGDY nanosheets. HAADF‐STEM (Figure 3) and High‐resolution TEM (HRTEM, Figure S4, Supporting Information) images verified no obvious Pt clusters and nanoparticles existed on the NGDY surface. Inductively coupled plasma mass spectrometry (ICP‐MS) analysis showed the Pt mass loading is 0.02 mg cm^–2^. The HAADF‐STEM‐EDS elemental mapping results (Figure 3e–h) of C, N, and Pt reveal the uniform dispersion of the Pt atoms.

X‐ray absorption near‐edge structure (XANES), extended X‐ray absorption fine structure spectroscopy (EXAFS) and XPS analysis provide the information of the electronic states of Pt atoms in the catalysts. Figure 3i displays the XANES spectra of Pt/NGDY and Pt foil at Pt L3 edge. Compared with Pt foil, Pt/NGDY gives an obvious negative shift in the energy, indicating that Pt atoms in Pt/NGDY are zero‐valent. This was also confirmed by the derivative XANES results (Figure S5, Supporting Information). The local structure environment and atomic dispersion of Pt was examined by EXAFS Fourier transforms (Figure 3j). For Pt foil, the peak at ≈2.6 Å was observed in Fourier‐transformed EXAFS (FT‐EXAFS), which attributed to the Pt—Pt bonding and were not detected in Pt/NGDY. This corresponds to HAADF‐STEM results and verifying the isolated existence of Pt single atoms in Pt/NGDY. While, in Pt/NGDY sample, the two peaks at around 1.5 Å correspond to the scattering interaction between Pt atoms and the first coordination shell of Pt‐N. The peak appears at 2.0 Å for Pt/NGDY, which is larger than Pt–N distance, corresponds to the Pt—C bond length. The subsequent quantitative EXAFS curve‐fitting analysis shows that the coordination number of N atoms in the first coordination sphere was estimated to be 3 at the distance of 1.6 Å, indicating a square‐pyramidal configuration for the Pt—N/O bonding. In addition, the coordination sphere of C atoms exhibits the coordination number of 7 at 2.2 Å (Table S1, Supporting Information). These results solidly demonstrate the success fully anchoring of isolated zero‐valent Pt atoms on NGDY.^[^ ^8^, ^9^ ^]^

The chemical states of N and C in Pt/NGDY were studied by XPS spectra. Pt 4f spectrum of Pt/NGDY (Figure 3k) located at 71.6 (Pt 4f_7/2_) and 74.9 eV (Pt 4f_5/2_), respectively, confirm that the Pt atoms on NGDY are mainly in zero‐valence,^[^ ^6^ ^]^ consistent with the XANES results (Figure 3i). In N 1s spectra of NGDY shows two different peaks (Figure 3l), at 400.28 and 399.05 eV, indicating the formation of aromatic pyrazole units in GDY. These types of N offer new anchoring sites for Pt atoms.^[^ ^10^, ^11^ ^]^ After the Pt atom anchoring, the N 1s binding energy shifts positively by 0.15 eV and the C 1s binding energy of Pt/NGDY shows a negative shift by 0.3 eV (Figure S6, Supporting Information). The D/G band ratio value of Pt/NGDY in Raman increased from 0.77 to 0.85, indicating the Pt/NGDY have more defects (Figure S7, Supporting Information). These results confirm the presence of interactions between NGDY and Pt atoms, as well as the obvious electron transfer between Pt atoms and C/N atoms, which are critical and beneficial for enhancing the catalytic activity, long‐term stability, and carbon monoxide resistance.

### 2.1 MOR Performances of Pt/NGDY

**Figure 4:**
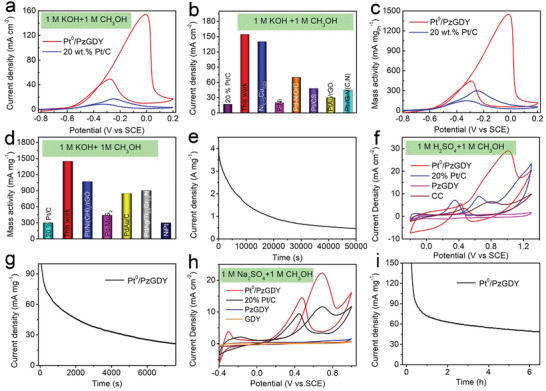
Electrocatalytic performances of Pt/NGDY for MOR. a) The CV curves of Pt/NGDY and commercial 20 wt% Pt/C. b) The current densities of Pt/NGDY and previously reported electrocatalysts. c,d) The mass activities of Pt/NGDY and previously reported electrocatalysts in alkaline conditions. e) The Pt/NGDY of long‐term durability measurements at 1 m methanol/1 m KOH. f) The current densities of these samples at 1 m methanol/1 m H_2_SO_4_. g) The Pt/NGDY of long‐term durability measurements at 1 m methanol/1 m H_2_SO_4_. h) The CV curves of these samples at 1 m methanol/1 m Na_2_SO_4_. i) The Pt/NGDY of long‐term durability measurements at 1 m methanol/1 m Na_2_SO_4_.

The electrocatalytic MOR performances of the Pt/NGDY, NGDY and commercial 20 wt% Pt/C were evaluated by using the cyclic voltammetry (CV) method at a sweep rate of 50 mV s^−1^ on a typical three‐electrode system at room temperatures. The electrochemical surface area (ECSA) with respect to the charge involved in hydrogen desorption for Pt/NGDY was determined to be 69.5 m^2^ g_Pt_^−1^, much larger than reported electrocatalysts^[^ ^14^ ^]^ (Figure S8, Supporting Information). The MOR tests were first performed in an aqueous solution of 1 m methanol and 1 m KOH. The specific activity curves normalized by corresponding surface area (**Figure** **4**a) reveal that Pt/NGDY has better MOR activity, with the larger current densities over the whole oxidation potentials, than that of Pt/C. For example, Pt/NGDY exhibits the largest current density of 154.2 mA cm^−2^, which is about 67 and 9 times larger than that of NGDY (2.3 mA cm^−2^) and commercial 20 wt% Pt/C (17.1 mA cm^−2^), respectively. Besides, Pt/NGDY exhibited a more negative onset potential (−0.6 V), as compared to Pt/C (−0.4 V), indicating that Pt/NGDY needs lower energy to drive the MOR than Pt/C. These results demonstrate the high intrinsic activity of Pt/NGDY. Such excellent specific activity is even better than previously reported Pt‐based electrocatalysts and other ones (Figure 4b, Table S2, Supporting Information). The mass activities (MOR current normalized by loading mass of Pt) of the samples were further obtained and shown in Figure 4c. As expected, Pt/NGDY possesses a significantly higher mass activity 1449 mA mg_Pt_^−1^ than commercial 20 wt% Pt/C (300 mA mg_Pt_^−1^) and previously reported Pt‐based electrocatalysts in alkaline conditions (Figure 4d). The operation stability of the Pt/NGDY is determined by the chronoamperometry test (CAT) at room temperature in 1 m CH_3_OH+1 m KOH aqueous solution. As shown in Figure 4e, Pt/NGDY shows much better stability with the current density retention of >74% for a 50 000 s continuous operation than that of 20 wt% Pt/C only 43% retention (Figure S9, Supporting Information), higher than that of 20 wt% Pt/C (43%). The current loss of Pt/NGDY may be due to the adsorption of methanol molecules which lead to the poisoning of the catalysts. HAADF characterizations on the Pt/NGDY samples after the long‐term stability test show that the Pt atoms remain isolated dispersion on the surface of NGDY without any aggregation and all elements are uniformly dispersed in NGDY (Figure S10 and S11, Supporting Information). The specific activity of Pt/NGDY was found to change with the variation of the Pt loadings. As the deposition time was further increased, Pt clusters and Pt nanoparticles could be observed, and the specific activity of Pt/NGDY decreased (Figure S12, Supporting Information). These results reveal that the suitable loading Pt in Pt/NGDY can lead to greater enhancement of activity and durability.

The MOR activities of Pt/NGDY were determined in 0.5 m H_2_SO_4_ and 1 m methanol electrolytes. As shown in Figure 4f, the Pt/NGDY displays the highest peak current density of 29 mA cm^–2^ than that of Pt/C, NGDY and CC. The stability of Pt/NGDY was examined Pt/NGDY at −0.65V. It was observed that the highest peak current density still retains 21 mA mg_Pt_^–1^ even after 7600 s (Figure 4g). The mass activity of Pt/NGDY (296 mA mg_Pt_^–1^) is larger than Pt/C (10 mA mg_Pt_^–1^) (Figure S13, Supporting Information). We next investigated the MOR performance and long‐term stability of the samples in 1 m Na_2_SO_4_ and 1 m methanol electrolytes. As expected, the Pt/NGDY still possesses a higher MOR activity with a current density of 22 mA cm^–2^ (110 mA mg_Pt_^–1^) than that of the Pt/C (current density of 11 mA cm^–2^, and mass activity of 14 mA mg_Pt_^–1^) (Figure 4h and Figure S14, Supporting Information). After a continuous electrocatalysis, the mass activity of Pt/NGDY retained 48 mA mg_Pt_^–1^ (Figure 4i). These findings demonstrate that the N doped GDY‐based zero‐valent Pt atomic catalysts can not only maximize the catalytic activity but also improve the long‐term stability.

**Figure 5:**
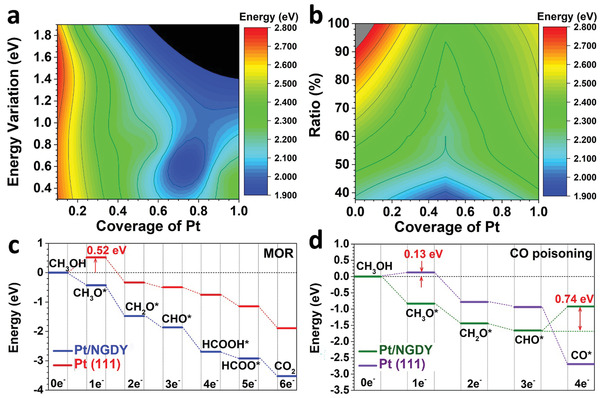
Theoretical studies. a) The energy mapping based on the Pt coverage and energy variation for Pt/NGDY. b) The energy mapping based on the Pt coverage and the ratio of the most stable Pt anchoring sites for Pt/NGDY. c)The energetic pathway of MOR on Pt/NGDY. d) The energetic pathway of CO poisoning during the MOR on Pt/NGDY.

The anti‐CO poisoning ability is another important indicator for evaluating the performance of a catalyst. The anti‐poisoning ability to carbonaceous species of the catalysts could be evaluated by quantitatively comparing the ratio of the peak current densities in the forward scan (*j*_f_) to backward scan (*j*_b_), the anti‐poisoning ability to carbonaceous species of the catalysts could be assessed.^[^ ^12^, ^13^, ^14^ ^]^ As expected, the *j*_f_/*j*_b_ ratio values of Pt/NGDY are 4.2, 1.63 and 1.26‐fold larger than the commercial 20 wt% Pt/C in acidic, alkaline and neutral solutions, revealing the better anti‐CO poisoning ability of Pt/NGDY than commercial 20 wt% Pt/C and implied the methanol would be effectively oxidized. This was also confirmed by the CO stripping voltammetry experiments. As shown in Figure S15 (Supporting Information), commercial 20 wt% Pt/C presents the oxidation peak at −0.51 V (vs SCE) due to the electrooxidation of CO on Pt/C.^[^ ^14^ ^]^ Remarkably, Pt/NGDY shows the oxidation peak started at a more negative potential of around −0.64 V (**Figure** **5**j), giving a 130 mV decrease in the onset potential compared to Pt/C. These results demonstrate the excellent MOR performance of Pt/NGDY in a wide pH range could be corresponding to the catalysts can efficiently reduce the CO binding strength, inconsistent with DFT calculations.

We further study the high coverage of Pt/NGDY from the energetic mapping. For the initial coverage of Pt on NGDY, the energy variation is very large due to the distinct energy cost of substantial potential anchoring sites (Figure 5a). As the coverage increases, the energy variation becomes more stable and reaches the smallest point at the coverage of ≈70%, indicating the limits for the most stable structures of high Pt coverage. This is due to the higher coverage that leads to the more even distribution of Pt atoms, lowering the overall energy. When the coverage further increases to larger than 70%, the additional Pt atoms occupy the more unstable, leading to the holistically unstable structure of Pt/NGDY. For the different Pt coverage structures, the ratio of the most energetically preferred anchoring sites also correlates with the stability of the catalyst (Figure 5b). As the Pt atoms start covering the NGDY, Pt atoms prefer to occupy the most stable anchoring site first. When the most stable positions are occupied, the occupation of Pt on other less stable anchoring sites results in the overall increases of the structure. Thus, the high coverage of the Pt on NGDY is achieved based on the balance between the Pt atoms distribution and stability. In addition, the energetic reaction pathways have been demonstrated for both MOR and CO poisoning (Figure 5c). For the MOR process, the overall reaction process exhibits the exothermal trend with an energy release of 3.52 eV, which supports a much stronger thermodynamic trend than the pristine Pt (111) surface. Such an energetically favorable trend is ascribed to the enhanced Pt coverage with high electroactivity to facilitate the electron transfer efficiency and intermediate reactions. Meanwhile, the Pt (111) surface demonstrates an evident barrier of 0.52 eV for the initial cleavage of the C—H bond, which significantly lowers the MOR efficiency. Meanwhile, the pristine Pt catalyst usually suffers from CO poisoning, which is a key factor to influencing the MOR performances (Figure 5d). Although the initial dehydrogenation of methanol requires a subtle barrier of 0.13 eV, the Pt (111) surface display an energetically favorable trend for the CO formation, leading to the inevitable occurrence of catalyst poisoning. In comparison, the high coverage of Pt atom on NGDY substantially suppresses the CO poisoning effect. Attributed to the varied Pt active sites on Pt/NGDY, the multi‐Pt sites are able to display stronger suppression effect to prevent the CO poisoning than the mono Pt atom anchoring on GDY or the pristine Pt catalyst. Such an integrated structure causes a high energy barrier of 0.74 eV to form CO from dehydrogenation of CHO* on Pt/NGDY, determining the high performance of MOR and durability of the Pt/NGDY.

## 3 Conclusion

In summary, we have demonstrated the atomic catalyst of Pt/NGDY is an almost perfect catalyst, especially as an efficient catalyst in MOR reaction. The dependent Pt/NGDY catalyst exhibits superior performance both high specific activity and mass activity in wide pH conditions. For example, the mass activity (specific activity) of Pt/NGDY toward MOR in alkaline, acidic, and neutral conditions are 1449 mA mg_Pt_^−1^ (154 mA cm^−2^), 296 mA mg_Pt_^–1^ (29 mA cm^−2^) and 110 mA mg_Pt_^–1^ (22 mA cm^−2^), which are 5, 29, and 8 times higher than Pt/C, respectively. And the Pt/NGDY catalysts also exhibited robust stability and resistant CO poisoning in MOR. Experimental and theoretical results reveal that the high Pt loading on NGDY is achieved due to the loss of symmetry and electronic homogenous in NGDY, which allows more Pt anchoring on different sites. The synergistic contributions of different Pt sites with varied electroactivity not only promote the electron transfer towards intermediates for MOR but also improve the suppression of the CO poisoning for enhanced durability. This strategy provides an effective way for the synthesis of robust atom catalysts with high catalytic performance for MOR.

## Conflict of Interest

The authors declare no conflict of interest.

## Supporting information

Supporting Information

## Data Availability

The data that support the findings of this study are available from the corresponding author upon reasonable request.

## References

[1] A. Wang , J. Li , T. Zhang , Nat. Rev. Chem. 2018, 2, 65.

[2] H. Fei , J. Dong , Y. Feng , C. S. Allen , C. Wan , B. Volosskiy , M. Li , Z. Zhao , Y. Wang , H. Sun , P. An , W. Chen , Z. Guo , C. Lee , D. Chen , I. Shakir , M. Liu , T. Hu , Y. Li , A. I. Kirkland , X. Duan , Y. Huang , Nat. Catal. 2018, 1, 63.

[3] P. Liu , Y. Zhao , R. Qin , S. Mo , G. Chen , L. Gu , D. M. Chevrier , P. Zhang , Q. Guo , D. Zang , B. Wu , G. Fu , N. Zheng , Science 2016, 352, 797. 27174982 10.1126/science.aaf5251

[4] J. Jones , H. Xiong , A. T. DeLaRiva , E. J. Peterson , H. Pham , S. R. Challa , G. Qi , S. Oh , M. H. Wiebenga , X. I. Pereira Hernández , Y. Wang , A. K. Datye , Science 2016, 353, 150. 27387946 10.1126/science.aaf8800

[5] B. Qiao , A. Wang , X. Yang , L. F. Allard , Z. Jiang , Y. Cui , J. Liu , J. Li , T. Zhang , Nat. Chem. 2011, 3, 634. 21778984 10.1038/nchem.1095

[6] Y. Ma , J. Lin , X.‐N. Song , C.‐K. Wang , W. Hua , Y. Luo , Carbon 2019, 149, 672.

[7] Y. Wang , H. Su , Y. He , L. Li , S. Zhu , H. Shen , P. Xie , X. Fu , G. Zhou , C. Feng , D. Zhao , F. Xiao , X. Zhu , Y. Zeng , M. Shao , S. Chen , G. Wu , J. Zeng , C. Wang , Chem. Rev. 2020, 120, 12217. 33136387 10.1021/acs.chemrev.0c00594

[8] L. Liu , A. Corma , Nat. Rev. Mater. 2020, 6, 244.

[9] C. Zhao , G.‐L. Xu , Z. Yu , L. Zhang , I. Hwang , Y.‐X. Mo , Y. Ren , L. Cheng , C.‐J. Sun , Y. Ren , X. Zuo , J.‐T. Li , S.‐G. Sun , K. Amine , T. Zhao , Nat. Nanotechnol. 2020, 16, 166. 33230316 10.1038/s41565-020-00797-w

[10] X. Li , L. Liu , X. Ren , J. Gao , Y. Huang , B. Liu , Sci. Adv. 2020, 6, 6833. 10.1126/sciadv.abb6833 PMC7531890 32967833

[11] T. Sun , S. Mitchell , J. Li , P. Lyu , X. Wu , J. Pérez‐Ramírez , J. Lu , Adv. Mater. 2021, 33, 2003075. 10.1002/adma.202003075 33283369

[12] B. Lu , Q. Liu , S. Chen , ACS Catal. 2020, 10, 7584.

[13] Z. Li , Y. Chen , S. Ji , Y. Tang , W. Chen , A. Li , J. Zhao , Y. Xiong , Y. Wu , Y. Gong , T. Yao , W. Liu , L. Zheng , J. Dong , Y. Wang , Z. Zhuang , W. Xing , C.‐T. He , C. Peng , W.‐C. Cheong , Q. Li , M. Zhang , Z. Chen , N. Fu , X. Gao , W. Zhu , J. Wan , J. Zhang , L. Gu , S. Wei , Nat. Chem. 2020, 12, 764. 32541950 10.1038/s41557-020-0473-9

[14] W. J. Huang , H. T. Wang , J. G. Zhou , J. Wang , P. N. Duchesne , D. Muir , P. Zhang , N. Han , F. P. Zhao , M. Zeng , J. Zhong , C. H. Jin , Y. G. Li , S. T. Lee , H. J. Dai , Nat. Commun. 2015, 6, 10035. 26602295 10.1038/ncomms10035 PMC4674678

[15] M. M. Liu , R. Z. Zhang , W. Chen , Chem. Rev. 2014, 114, 5117. 24666160 10.1021/cr400523y

[16] T. T. Sun , S. Zhao , W. X. Chen , D. Zhai , J. C. Dong , Y. Wang , S. L. Zhang , A. J. Han , L. Gu , R. Yu , X. D. Wen , H. L. Ren , L. B. Xu , C. Chen , Q. Peng , D. S. Wang , Y. D. Li , Proc. Natl. Acad. Sci. U. S. A. 2018, 115, 12692. 30487213 10.1073/pnas.1813605115 PMC6294881

[17] L. Huang , X. Zhang , Q. Wang , Y. Han , Y. Fang , S. Dong , J. Am. Chem. Soc. 2018, 140, 1142. 29283565 10.1021/jacs.7b12353

[18] S. J. Guo , S. Zhang , X. L. Sun , S. H. Sun , J. Am. Chem. Soc. 2011, 133, 15354. 21894999 10.1021/ja207308b

[19] Z. C. Zhang , Z. M. Luo , B. Chen , C. Wei , L. Zhao , J. Z. Chen , X. Zhang , Z. C. Lai , Z. X. Fan , C. L. Tan , M. T. Zhao , Q. P. Lu , B. Li , Y. Zong , C. C. Yan , G. X. Wang , Z. J. C. Xu , H. Zhang , Adv. Mater. 2016, 28, 8712. 27511958 10.1002/adma.201603075

[20] L. Huang , J. S. Zou , J. Y. Ye , Z. Y. Zhou , Z. Lin , X. W. Kang , P. K. Jain , S. W. Chen , Angew. Chem., Int. Ed. 2019, 58, 8794. 10.1002/anie.201903290 31038831

[21] Y. Liu , F. Chen , Q. Wang , J. Wang , J. Wang , L. Guo , T. T. Gebremariam , Nanoscale 2019, 11, 8812. 31011725 10.1039/c9nr00361d

[22] G. X. Li , Y. L. Li , H. B. Liu , Y. B. Guo , Y. J. Li , D. B. Zhu , Chem. Commun. 2010, 46, 3256. 10.1039/b922733d 20442882

[23] Y. S. Zhao , J. W. Wan , H. Y. Yao , L. J. Zhang , K. F. Lin , L. Wang , N. L. Yang , D. B. Liu , L. Song , J. Zhu , L. Gu , L. Liu , H. J. Zhao , Y. L. Li , D. Wang , Nat. Chem. 2018, 10, 924. 30082882 10.1038/s41557-018-0100-1

[24] J. J. He , N. Wang , Z. L. Cui , H. P. Du , L. Fu , C. S. Huang , Z. Yang , X. Y. Shen , Y. P. Yi , Z. Y. Tu , Y. L. Li , Nat. Commun. 2017, 8, 1172. 29079826 10.1038/s41467-017-01202-2 PMC5660080

[25] C. Y. Xing , Y. R. Xue , B. L. Huang , H. D. Yu , L. Hui , Y. Fang , Y. X. Liu , Y. J. Zhao , Z. B. Li , Y. L. Li , Angew. Chem., Int. Ed. 2019, 58, 13897. 10.1002/anie.201905729 31309671

[26] N. Wang , J. J. He , Z. Y. Tu , Z. Yang , F. H. Zhao , X. D. Li , C. S. Huang , K. Wang , T. G. Jiu , Y. P. Yi , Y. L. Li , Angew. Chem., Int. Ed. 2017, 56, 10740. 10.1002/anie.201704779 28691245

[27] N. Wang , X. D. Li , Z. Y. Tu , F. H. Zhao , J. J. He , Z. Y. Guan , C. S. Huang , Y. P. Yi , Y. L. Li , Angew. Chem., Int. Ed. 2018, 57, 3968. 10.1002/anie.201800453 29397008

[28] Y. R. Xue , B. L. Huang , Y. P. Yi , Y. Guo , Z. C. Zuo , Y. J. Li , Z. Y. Jia , H. B. Liu , Y. L. Li , Nat. Commun. 2018, 9, 1460. 29654234 10.1038/s41467-018-03896-4 PMC5899097

[29] L. Hui , Y. R. Xue , H. D. Yu , Y. X. Liu , Y. Fang , C. Y. Xing , B. L. Huang , Y. L. Li , J. Am. Chem. Soc. 2019, 141, 10677. 31149825 10.1021/jacs.9b03004

[30] Y. Fang , Y. Xue , Y. Li , H. Yu , L. Hui , Y. Liu , C. Xing , C. Zhang , D. Zhang , Z. Wang , X. Chen , Y. Gao , B. Huang , Y. Li , Angew. Chem., Int. Ed. 2020, 132, 13121.

[31] Q. Yang , Y. Guo , B. X. Yan , C. D. Wang , Z. X. Liu , Z. D. Huang , Y. K. Wang , Y. R. Li , H. F. Li , L. Song , J. Fan , C. Y. Zhi , Adv. Mater. 2020, 32, 2001755. 10.1002/adma.202001755 32406976

[32] F. Wang , Z. C. Zuo , L. Li , K. Li , F. He , Z. Q. Jiang , Y. L. Li , Angew. Chem., Int. Ed. 2019, 58, 15010. 10.1002/anie.201910588 31478303

[33] H. Shang , Z. Zuo , L. Li , F. Wang , H. Liu , Y. Li , Y. Li , Angew. Chem., Int. Ed. 2018, 57, 774. 10.1002/anie.201711366 29181867

[34] B. Y. Xia , H. B. Wu , X. Wang , X. W. Lou , J. Am. Chem. Soc. 2012, 134, 13934. 22897642 10.1021/ja3051662

[35] K. P. Gong , F. Du , Z. H. Xia , M. Durstock , L. M. Dai , Science 2009, 323, 760. 19197058 10.1126/science.1168049

[36] Z. Li , Y. J. Chen , S. F. Ji , Y. Tang , W. X. Chen , A. Li , J. Zhao , Y. Xiong , Y. E. Wu , Y. Gong , T. Yao , W. Liu , L. R. Zheng , J. C. Dong , Y. Wang , Z. B. Zhuang , W. Xing , C. T. He , C. Peng , W. C. Cheong , Q. H. Li , M. L. Zhang , Z. Chen , N. H. Fu , X. Gao , W. Zhu , J. W. Wan , J. Zhang , L. Gu , S. Q. Wei , Nat. Chem. 2020,12, 764. 32541950 10.1038/s41557-020-0473-9

[37] D. H. Guo , R. Shibuya , C. Akiba , S. Saji , T. Kondo , J. Nakamura , Science 2016, 351, 361. 26798009 10.1126/science.aad0832

[38] T. Q. Lin , I. W. Chen , F. X. Liu , C. Y. Yang , H. Bi , F. F. Xu , F. Q. Huang , Science 2015, 350, 1508. 26680194 10.1126/science.aab3798

[39] A. Zitolo , V. Goellner , V. Armel , M. T. Sougrati , T. Mineva , L. Stievano , E. Fonda , F. Jaouen , Nat. Mater. 2015, 14, 937. 26259106 10.1038/nmat4367

[40] S. L. Zhang , H. P. Du , J. J. He , C. S. Huang , H. B. Liu , G. L. Cui , Y. L. Li , ACS. Appl. Mater. Interfaces 2016, 8, 8467. 26998614 10.1021/acsami.6b00255

[41] R. J. Liu , H. B. Liu , Y. L. Li , Y. P. Yi , X. K. Shang , S. S. Zhang , X. L. Yu , S. J. Zhang , H. B. Cao , G. J. Zhang , Nanoscale 2014, 6, 11336. 25141067 10.1039/c4nr03185g

[42] B. Y. Xia , H. B. Wu , X. Wang , X. W. Lou , J. Am. Chem. Soc. 2012, 134, 13934. 22897642 10.1021/ja3051662

[43] Z. M. Cui , H. Chen , M. T. Zhao , D. Marshall , Y. C. Yu , H. Abruna , F. J. DiSalvo , J. Am. Chem. Soc. 2014, 136, 10206. 25000137 10.1021/ja504573a

