# Supplementary material for: Highly Loaded Independent Pt0 Atoms on Graphdiyne for pH‐General Methanol Oxidation Reaction
Source: Adv Sci (Weinh). 2022 Apr 7;9(16):2104991. doi: 10.1002/advs.202104991 (PMC9165484; doi:10.1002/advs.202104991)
Supplement: Supplementary file 1 — Supporting Information [file ADVS-9-2104991-s001.pdf]

ADVANCED SCIENCE
Open Access

Supporting Information

for *Adv. Sci.*, DOI 10.1002/advs.202104991

Highly Loaded Independent Pt^0 Atoms on Graphdiyne for pH-General Methanol Oxidation Reaction

Lan Hui, Yurui Xue, Chengyu Xing, Yuxin Liu, Yuncheng Du, Yan Fang, Huidi Yu, Bolong Huang* and Yuliang Li**

Supporting Information

Highly loaded independent platinum atoms on graphdiyne for pH-general methanol oxidation reaction

Lan Hui, Yurui Xue, Chengyu Xing, Yuxin Liu, Yuncheng Du, Yan Fang, Huidi Yu, Bolong Huang* and Yuliang Li**

Experimential section

Synthesis of NGDY. GDY was immersed in a 50 mL Teflon lined autoclave containing H_2O (15 mL), $NH_3{\cdot}H_2O$ (5 mL) and $N_2H_4{\cdot}H_2O$ (5 mL) at 150 ^oC for 6 h. The obtained samples were thoroughly washed by deionized water and ethanol.

Synthesis of Pt/NGDY.

Pt/NGDY was prepared via electrochemical reduction method. 10 mg H_2PtCl_6 $\cdot 6H_2O$ was solved in 20 mL H_2SO_4 solution and blow Ar for 3h. Apieces of NGDY was at different times for loading Pt by electrochemical reduction treatment. The Pt/NGDY catalysts were washed by deionized water.

Characterizations. SEM images were conducted by S-4800 field emission scanning electron microscope. TEM/HRTEM were recorded by JEM-2100F transmission electron microscope. HAADF-STEM images were collected on an aberration-corrected cubed FET Titan Cubed Themis G2 300 or JEM-ARM200F (JEOL, Tokyo, Japan). The XRD of these samples were performed by Rigaku with Cu Kα radiation (λ = 0.15406 nm). The valence states and chemical composition of these samples were obtained by XPS measurements (Al Kα, hv =1486.6 eV). Raman spectra of these samples were carried out by Renishaw-2000 Raman spectrometer with 473 nm excitation laser source.

Calculation Setup

To investigate the electronic and energetic properties of Pt/NGDY for MOR, we choose the DFT calculations within the CASTEP code[1] for all the calculations in this work. We have selected the algorithm of Broyden-Fletcher-Goldfarb-Shannon (BFGS) to achieve ground state geometry optimization[2]. The GGA with the PBE exchange-correlation functionals are applied in this work[3-4]. The cutoff energy of plane-wave basis sets for total energy calculations has been set to 310 eV based on the ultrasoft pseudopotential[5].

The NGDY model is constructed based on the 2×2 GDY model, where the N dopants randomly occupy half of the GDY chains. The Pt coverage models of Pt/NGDY have been constructed based on 1 Pt atom to 10 Pt atoms on NGDY without any formation of the Pt-Pt bonds. All possible combinations of the Pt anchoring sites are considered for the energy mapping of the Pt coverage on NGDY. To ensure sufficient space for all the geometry optimizations and intermediate adsorptions, the vacuum space of 15 Å along the z-axis has been applied. Considering the DFT computational cost, the Monkhost-Pack reciprocal space integration was performed using Gamma-center-off special k-points with a mesh of 2×2×2 [6], which was guided by the initial convergence test. For the convergence of each step, the total energy and the Hellmann-Feynman forces on the atom should not exceed 5.0×10^{-5} eV per atom and 0.1 eV per angstrom, respectively.

XAFS measurements.

The X-ray absorption find structure spectra (Pt L3-edge) were collected at 1W1B station in Beijing Synchrotron Radiation Facility (BSRF). The storage rings of BSRF were operated at 2.5 GeV with an average current of 250 mA. Using Si (111) double-crystal monochromator, the data collection was carried out in transmission/fluorescence mode using ionization chamber. All spectra were collected in ambient conditions.

XAFS Analysis and Results.

The acquired EXAFS data were processed according to the standard procedures using the ATHENA module implemented in the IFEFFIT software packages. The k3-weighted EXAFS spectra were obtained by subtracting the post-edge background from the overall absorption and then normalizing with respect to the edge-jump step. Subsequently, k3-weighted $\chi(k)$ data of Fe Pt L3-edge were Fourier transformed to real (R) space using a hanning windows

(dk=1.0 Å-1) to separate the EXAFS contributions from different coordination shells. To obtain the quantitative structural parameters around central atoms, least-squares curve parameter fitting was performed using the ARTEMIS module of IFEFFIT software packages.

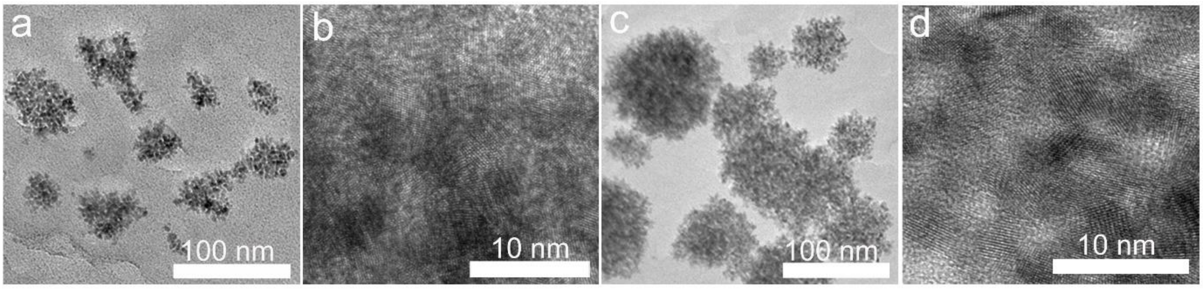

Figure S1. Low- and HR-TEM images of Pt/NGDY at (a,b) 20 s and (c,d) 40 s.

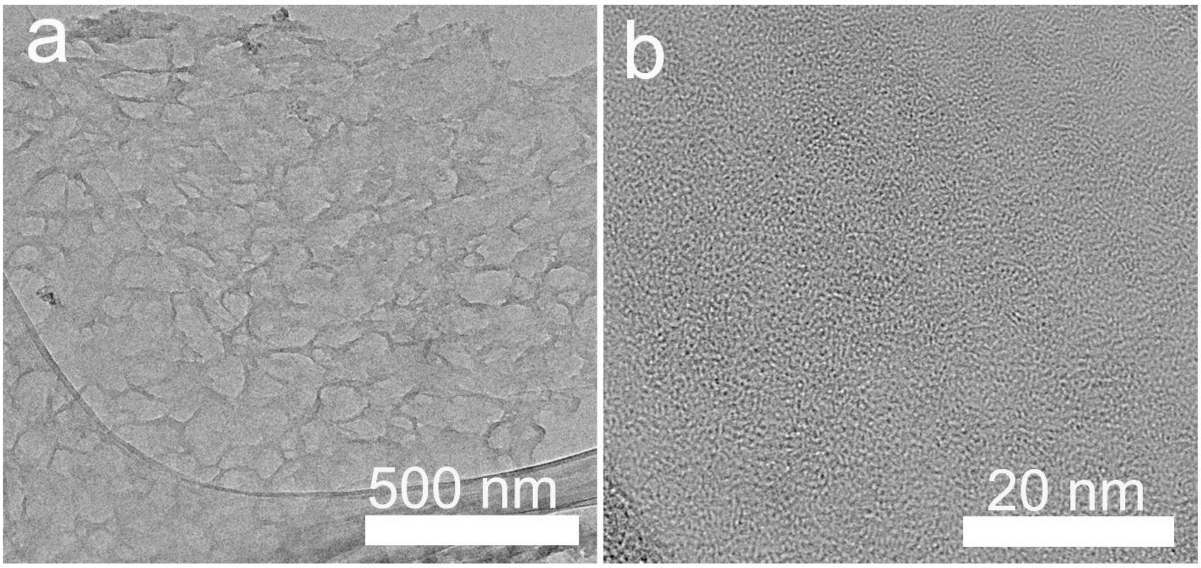

Figure S2. (a) Low- and (b) high-resolution TEM images of Pt/NGDY sample.

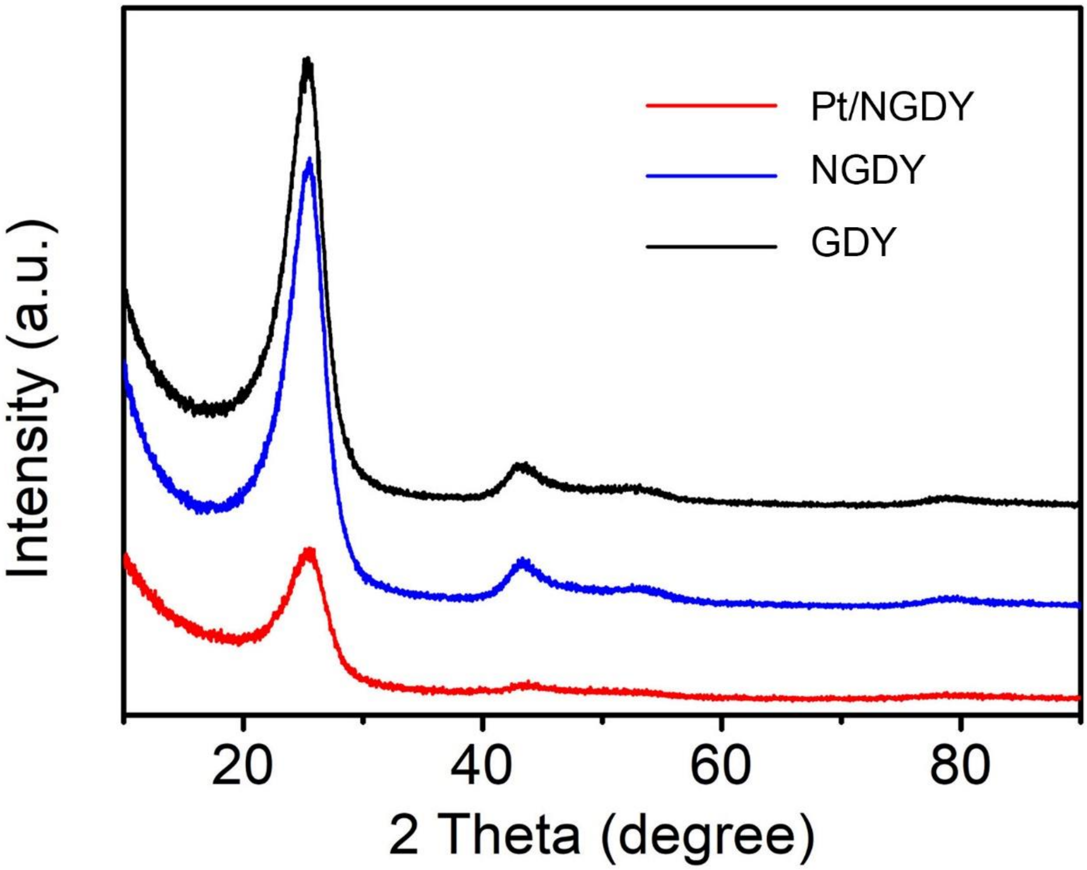

Figure S3. The XRD of Pt/NGDY (red line), NGDY (blue line) and GDY (black line).

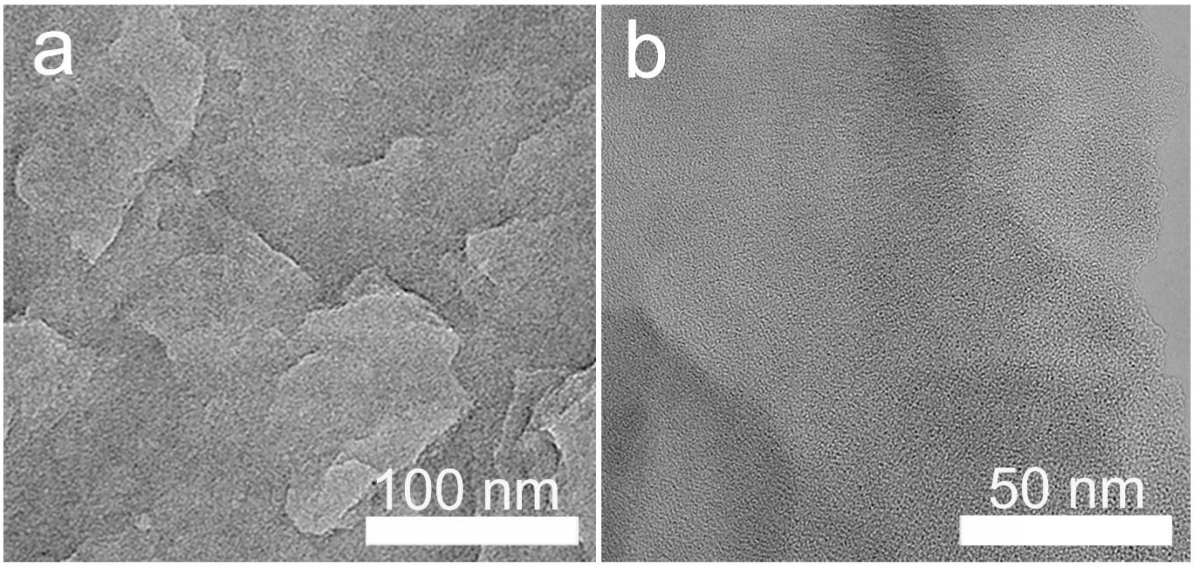

Figure S4. (a) Low- and (b) high-resolution TEM images of NGDY samples.

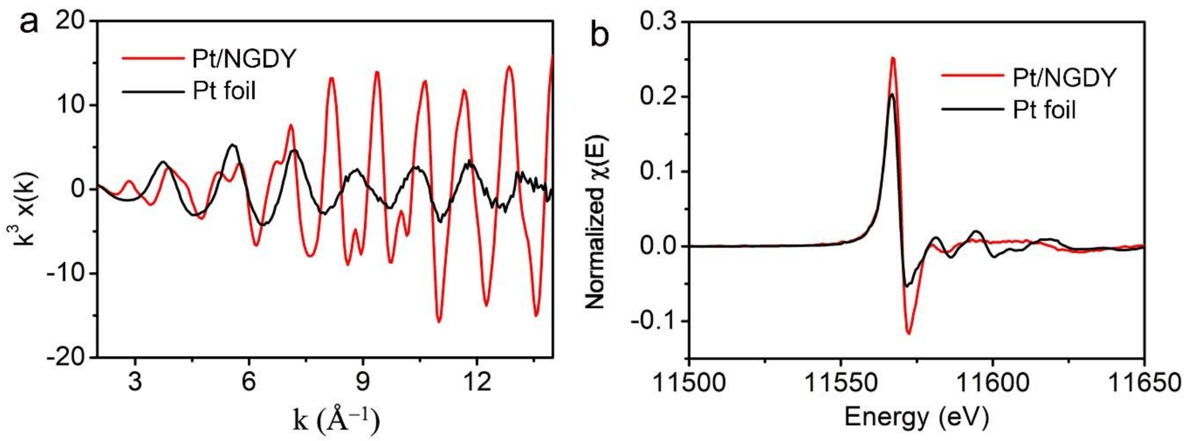

Figure S5. (a) The k space fitting curves of these samples. (b) The corresponding calculated first-derivative curves of these samples.

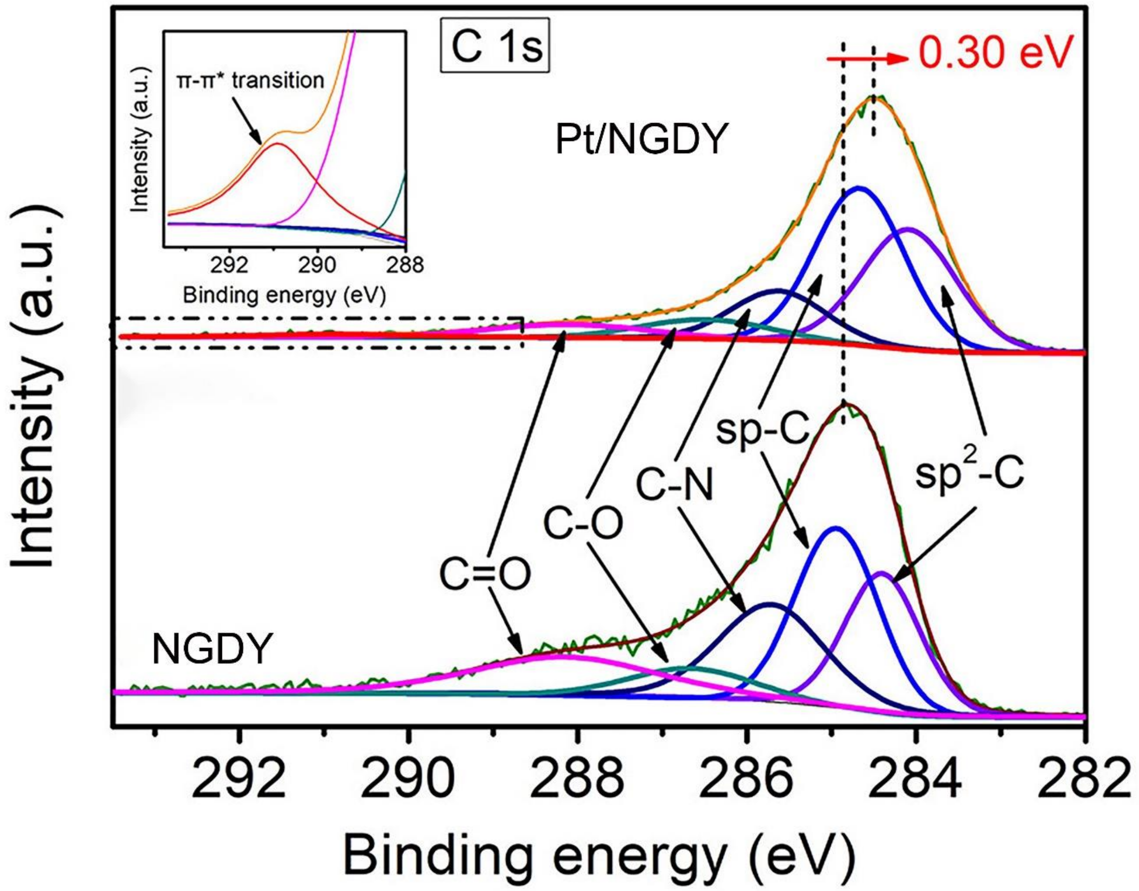

Figure S6. The N 1s XPS spectra of Pt/NGDY (upper) and NGDY (lower).

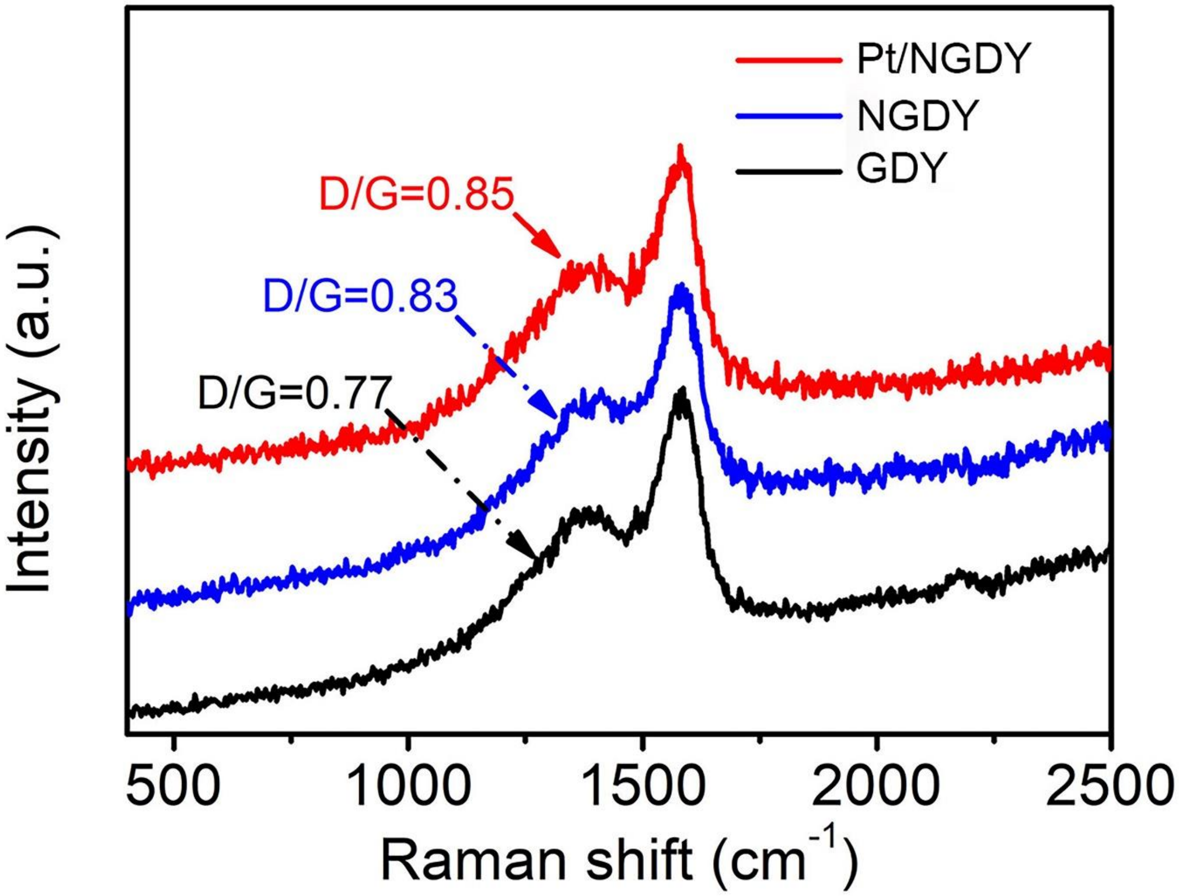

Figure S7. The Raman spectra of Pt/NGDY (red line), NGDY (blue line) and GDY (black line).

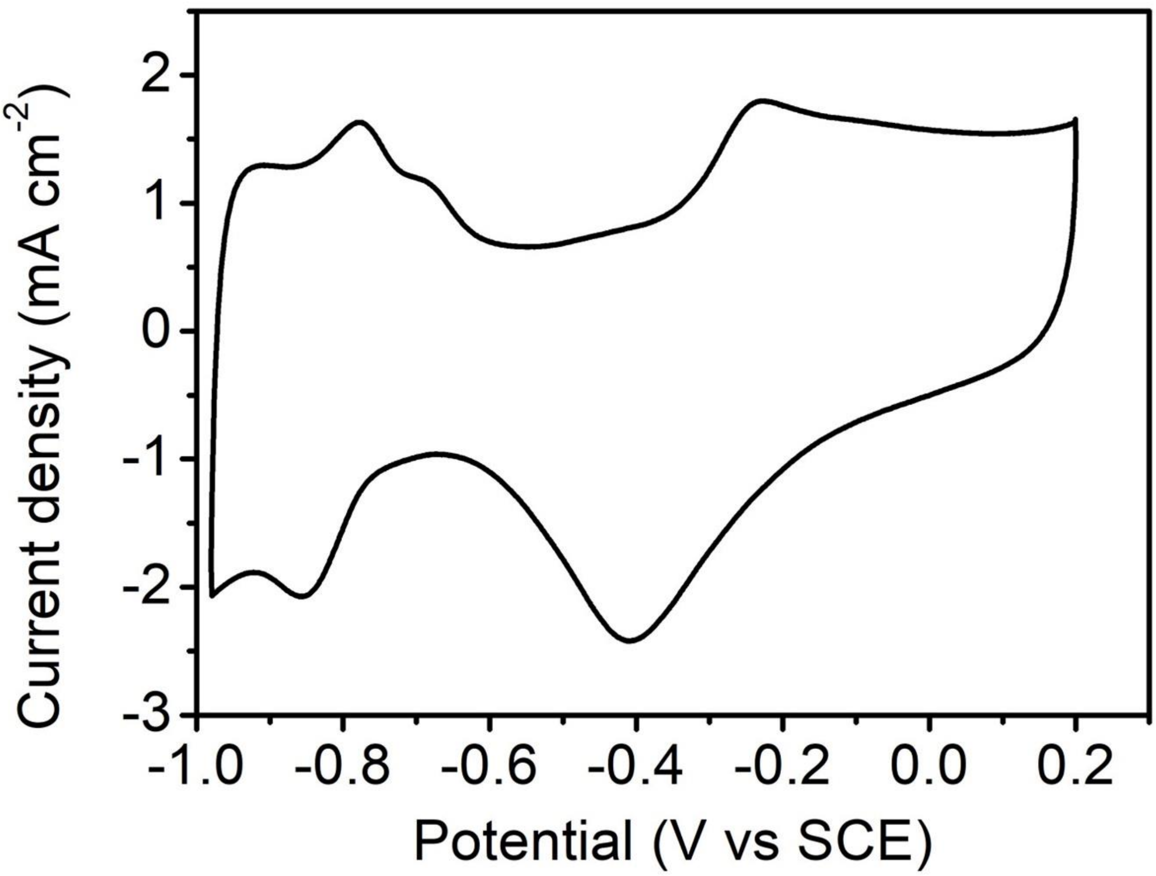

Figure S8. The ECSA curves of Pt/NGDY in N_2-alkaline solutions.

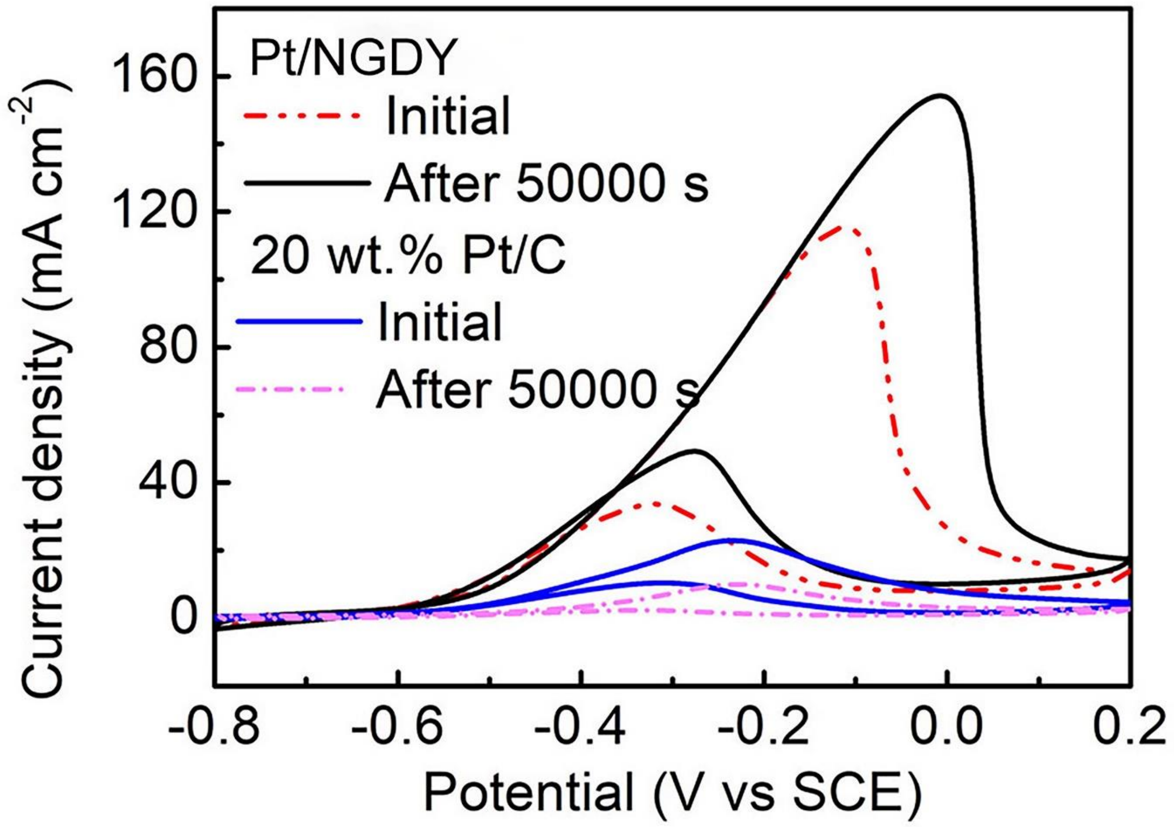

Figure S9. The CV curves of Pt/NGDY and commercial Pt/C retention after 50000 s in alkaline solution.

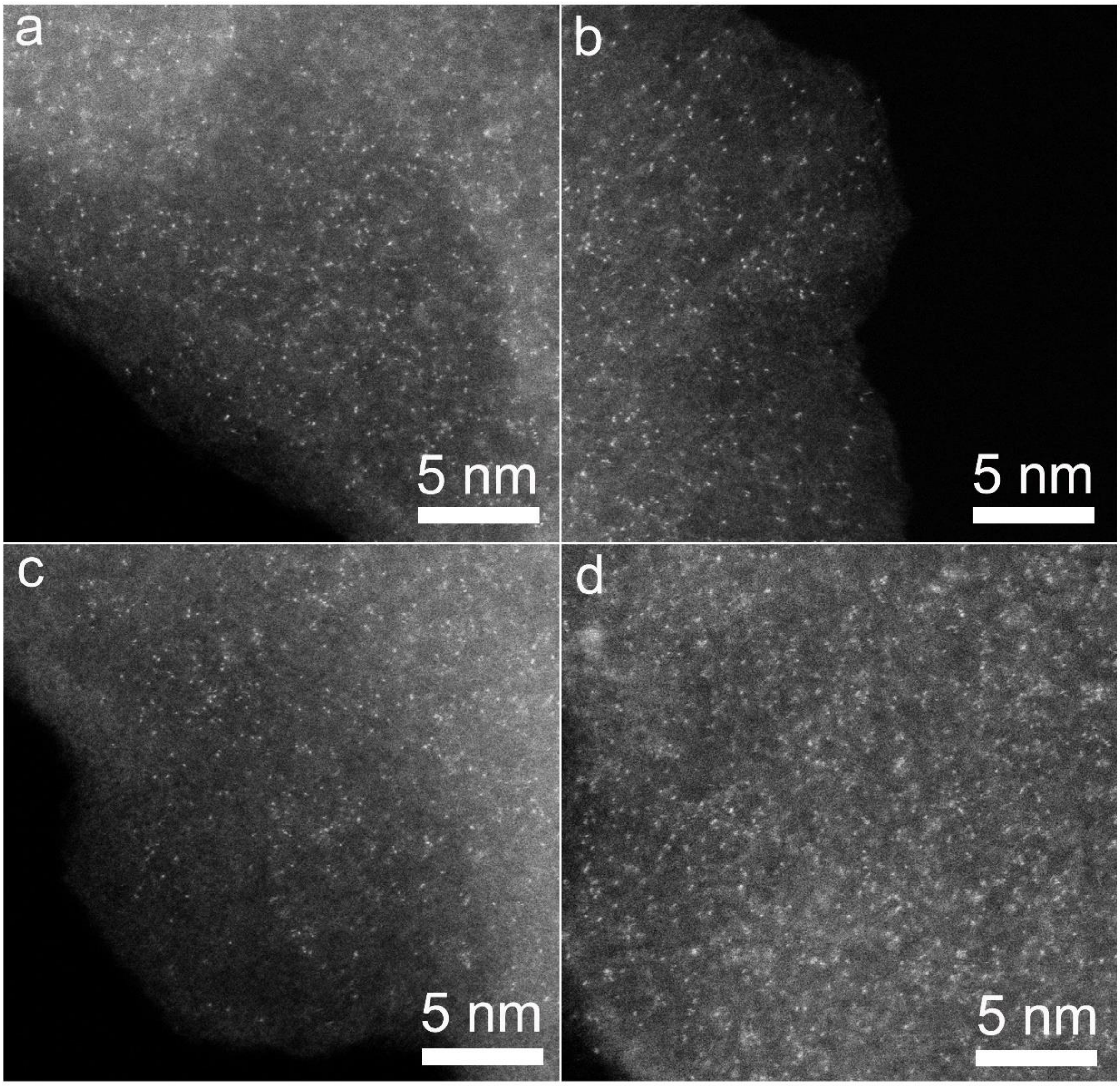

Figure S10. (a-d) HAADF images of Pt/NGDY after long-term stability.

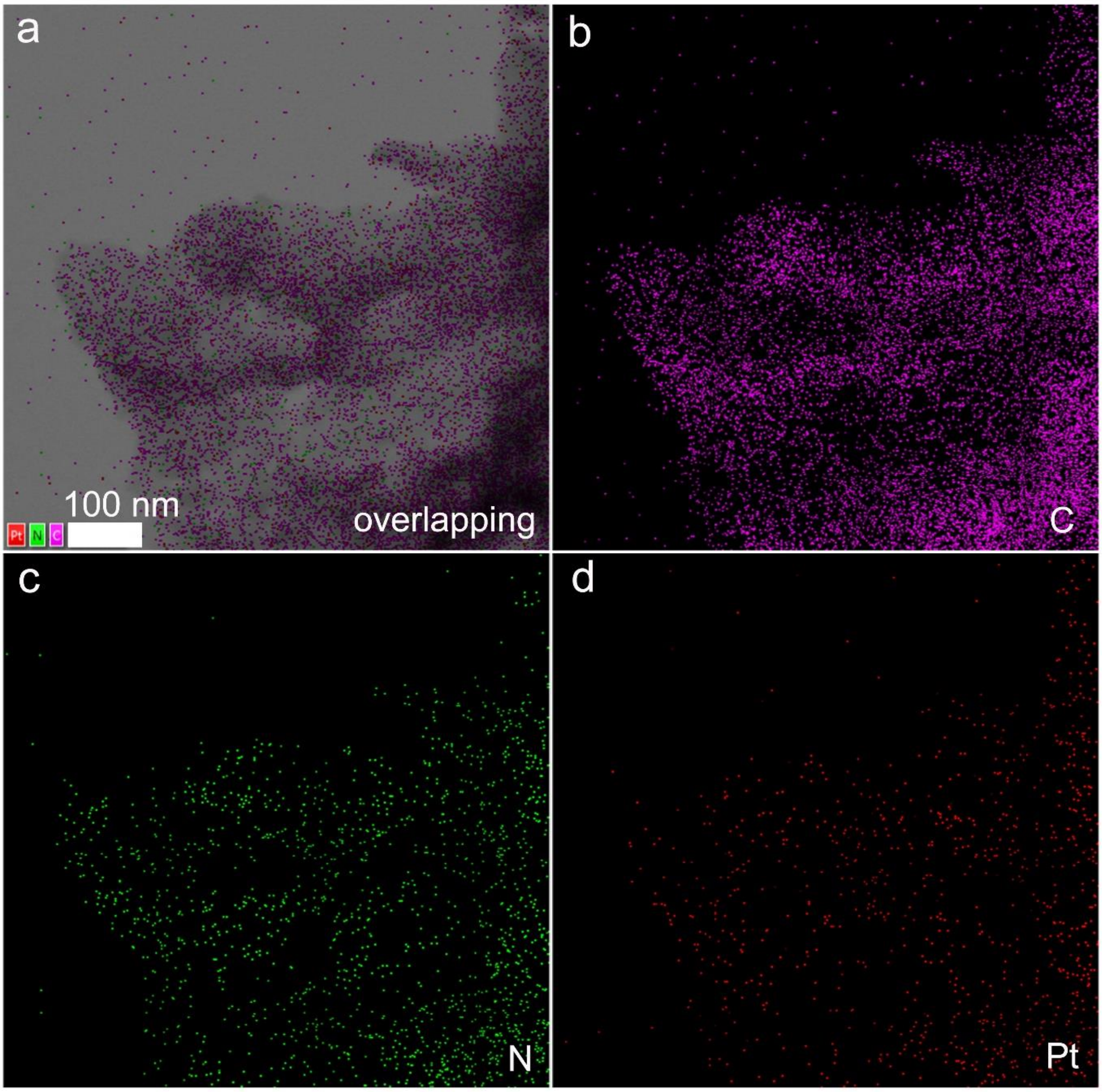

Figure S11. (a-d) EDX elemental mapping of images of Pt/NGDY after long-term stability.

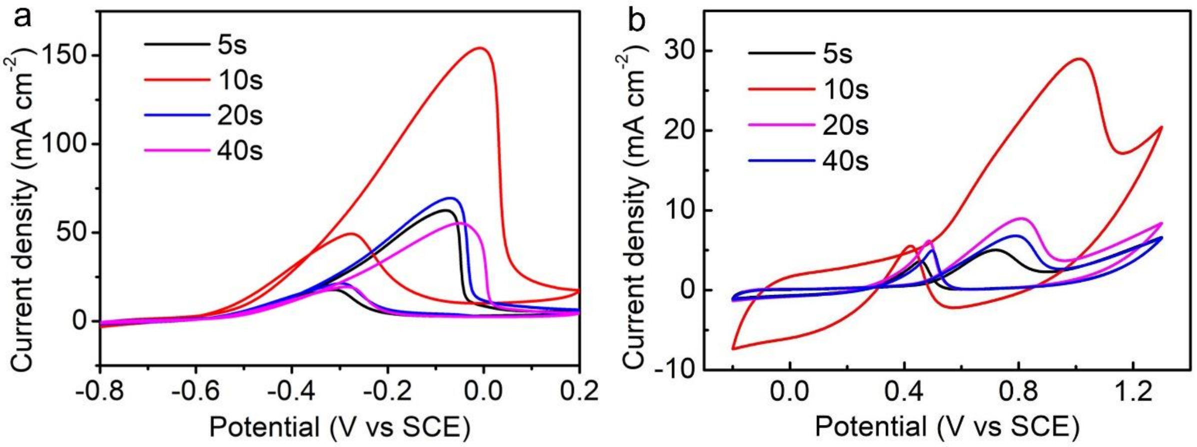

Figure S12. The MOR specific activity of Pt/NGDY samples with different Pt loadings in (a) 1 M KOH+1 M CH_3OH and (b) 1 M H_2SO_4+1 M CH_3OH solutions.

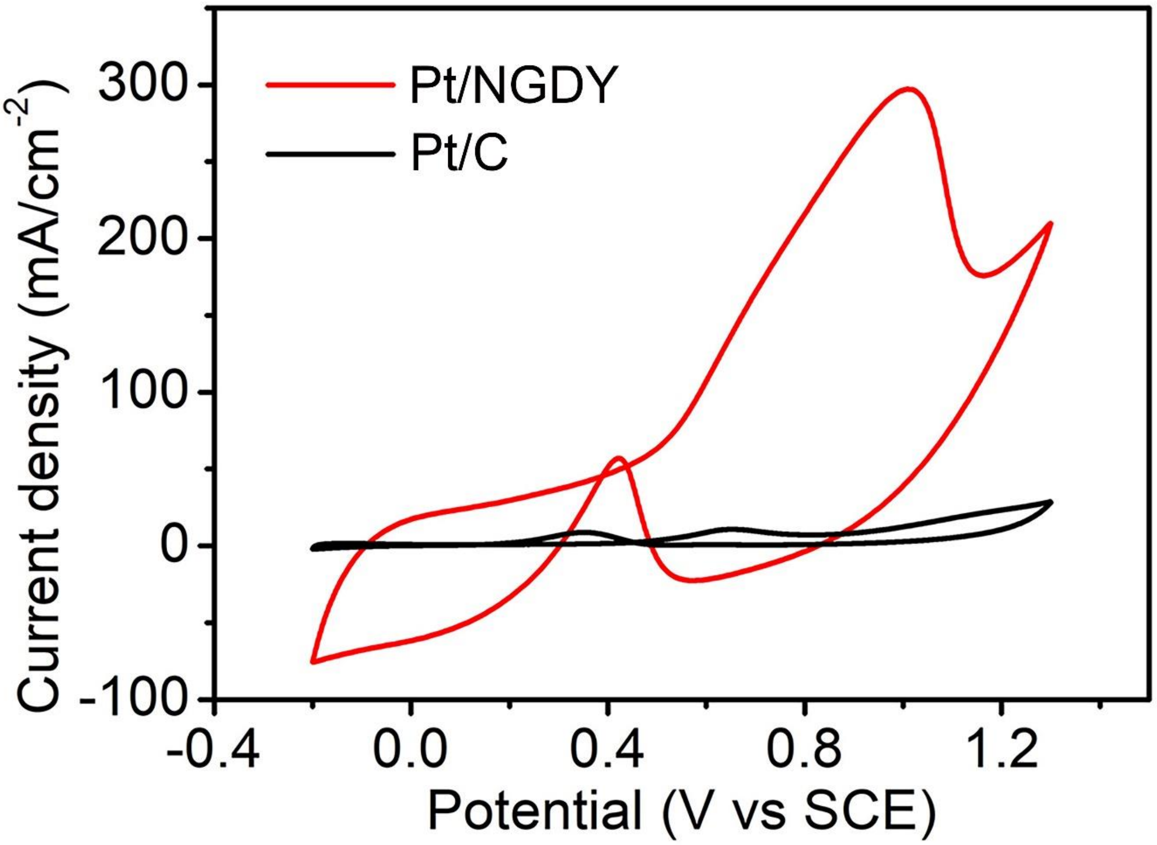

Figure S13. The mass activity of Pt/NGDY in 1 M H_2SO_4 + 1 M CH_3OH solutions.

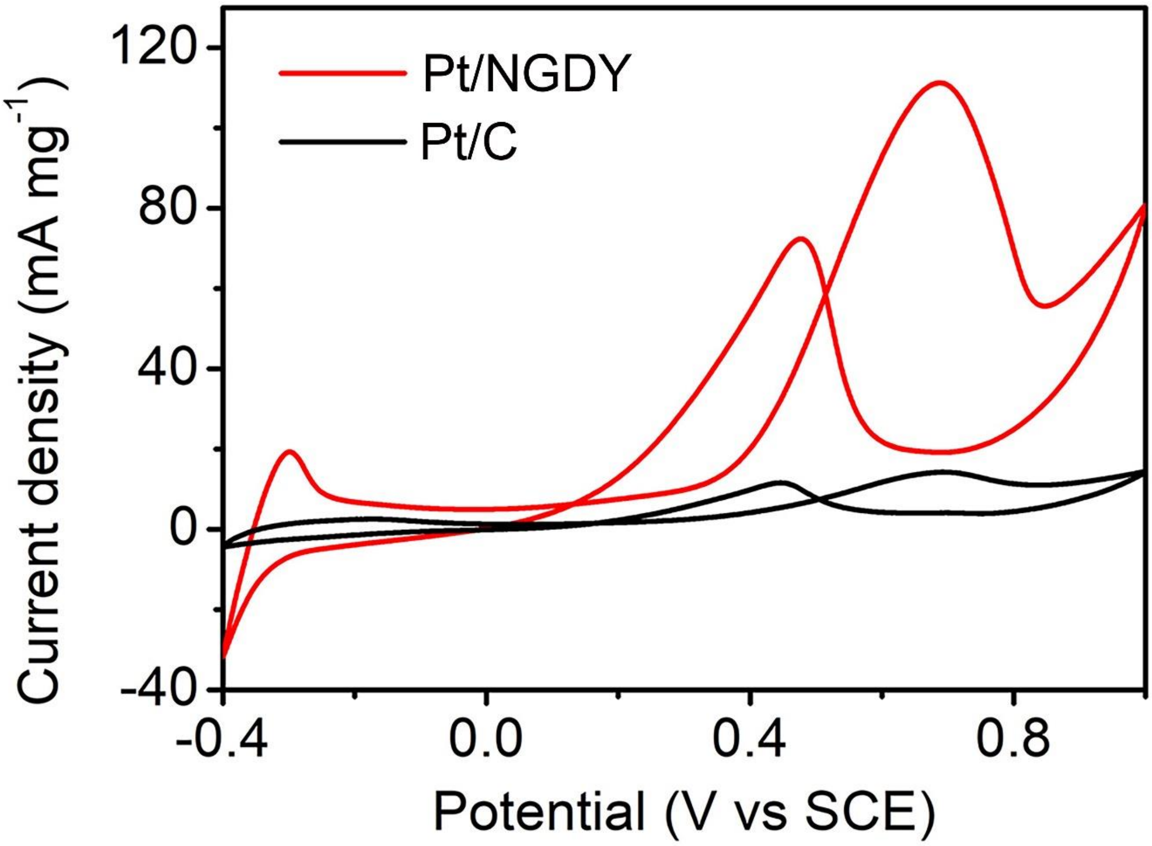

Figure S14. The mass activity of Pt/NGDY in 1 M Na_2SO_4 + 1 M CH_3OH solutions.

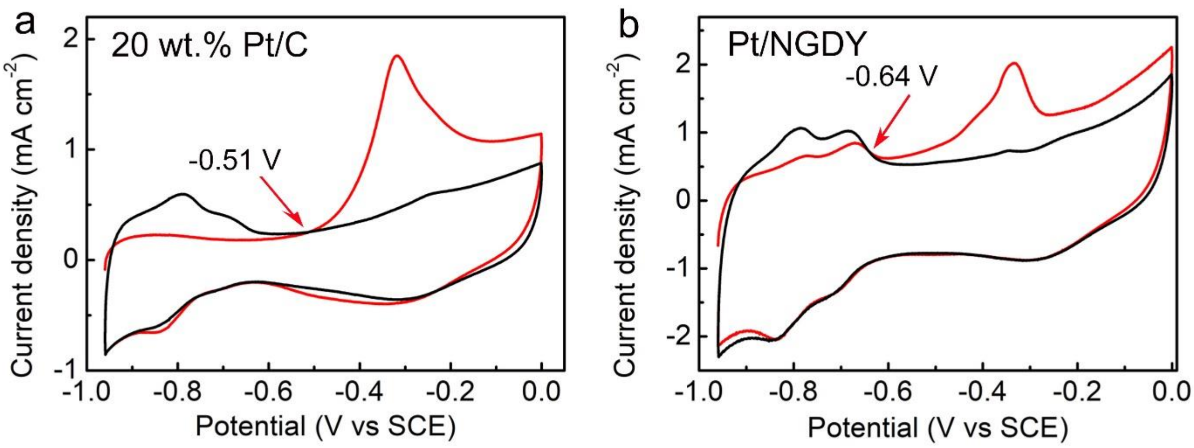

Figure S15. The CO poisoning of (a) commercial Pt/C and (b) Pt/NGDY.

Table S1. The average C/N/Pt ratio by HAADF-STEM-EDS.

Z	Element	family	Atomic Fraction (%)	Atomic Error (%)	Mass Fraction (%)	Mass Error (%)	Fit Error(%)
6	C	K	90.07	3.52	79.99	1.94	0.69
7	N	K	9.20	1.91	9.53	1.96	4.16
78	Pt	L	0.73	0.08	10.48	1.17	0.87

Table S2. Comparison of the current density and mass activity of Pt/NGDY electrocatalysts for MOR performances in 1 M KOH electrolytes with reported catalysts.

**Electrocatalyst**	**Electrolyte**	**Peak current from CV curves**	**Reference**
Pt/NGDY	1 M KOH + 1 M methanol<br>1 M H_2SO_4 + 1 M methanol<br>1 M Na_2SO_4 + 1 M methanol	1449 mA mg_{Pt}^{-1} (154 mA cm^{-2}),<br>296 mA mg_{Pt}^{-1} (29 mA cm^{-2})<br>110 mA mg_{Pt}^{-1} (22 mA cm^{-2})	This work
$Ni_{0.75}Cu_{0.25}$	1 M NaOH + 0.5 M methanol	140 mA cm^{-2}	*Angew. Chem. Int. Ed.* 2017, 56, 4488 –4493
Pt/Ni $(OH)_2$/rGO	1 M KOH + 1 M methanol	1.24 A mg^{-1} (150 mA cm^{-2})	*Nat. Commun.* 2015, 6, 10035
Pd/MoS_2	0.5 M KOH + 1 M methanol	440 mA mg_{Pt}^{-1}	*Nanoscale, 2014, 6, 5762-5769*
Pt /G-V (C, N)	1 M KOH + 0.5 M methanol	45 mA cm^{-2}	*Nanoscale.* 2015, 7, 1301-1307
$PdAg/Ti_{0.5}Cr_{0.5}N$	0.1 M KOH + 1 M methanol	0.84 A $mg^{-1}{}_{Pd}$	*ACS Nano.* 2014, 8, 6106-6113
PtNi/C	1 M NaOH + 1 M methanol	1.20 A mg^{-1}	*Catal. Commun.* 2010, 12, 67-70
PtAu/rGO	1 M KOH + 1 M methanol	30 mA cm^{-2}	*J. Mater. Chem. A,* 2014, 2, 8386
Pt/CS	1 M NaOH + 1 M methanol	48 mA cm^{-2}	*Mater. Lett.* 2013 106, 287-289
Pd-NiO/C	1 M KOH + 1 M methanol	70 mA cm^{-2}	*Electrochim. Acta,* 2013, 90, 108-111
PdAu/C	1 M KOH + 1 M methanol	0.90 A mg^{-1}	*J. Mater. Chem. A,* 2013, **1**, 9157

Table S3. EXAFS fitting parameters at the Pt L3-edge for various samples

Sample	Shell	N [a]	R (Å) [b]	σ^2 (Å^2·10^{-3}) [c]	ΔE0 (eV) [d]	*R* factor (%)
2	Pt-N	1.8	1.88	6.9	-10.8	0.3
	Pt-C	11.9	2.44	3.8	-7.8	

[a]*N*: coordination numbers; [b]*R*: bond distance; [c]σ^2: Debye-Waller factors; [d]ΔE_0: the inner potential correction. *R* factor: goodness of fit. *S*02 were set as 0.84/0.84 for Pt-O/Pt-C, which were obtained from the experimental EXAFS fit of reference PtO_2/Pt foil by fixing CN as the known crystallographic value and was fixed to all the samples[7-9].

References

(1) S. J. Clark, M. D. Segall, C. J. Pickard, P. J. Hasnip, M. I. J. Probert, K. Refson, M. C. Payne. **2005**, *220*, 567.

(2) J. D. Head, M. C. Zerner. Chem. Phys. Lett. **1985**, *122*, 264-270.

(3) J. P. Perdew, K. Burke, M. Ernzerhof. Phys. Rev. Lett. **1996**, *77*, 3865-3868.

(4) J. P. Perdew; M. Ernzerhof; K. Burke. J. Chem. Phys. **1996**, *105*, 9982-9985.

(5) P. J. Hasnip, C. J. Pickard. Comput. Phys. Commun. **2006**, *174*, 24-29.

(6) M. I. J. Probert, M. C. Payne. Phys. Rev. B **2003**, *67*, 075204.

(7) Ravel, B. & Newville, M. ATHENA, ARTEMIS, HEPHAESTUS: data analysis for X-ray absorption spectroscopy using IFEFFIT. J. Synchrotron Radiat. **2005**, *12*, 537-541.

(8) Koningsberger, D. C. & Prins, R. X-ray Absorption: Principles, Applications, Techniques of EXAFS, SEXAFS, and XANES (eds Koningsberger, D. C. & Prins, R.) Vol. 92 (Wiley, 1988).

(9) J. J. Rehr, R. C. Albers. Rev. Mod. Phys. **2000**, *72*, 621-654.
